# Estimating preferences for a dermatology consultation using Best-Worst Scaling: Comparison of various methods of analysis

**DOI:** 10.1186/1471-2288-8-76

**Published:** 2008-11-18

**Authors:** Terry N Flynn, Jordan J Louviere, Tim J Peters, Joanna Coast

**Affiliations:** 1 Department of Social Medicine, University of Bristol, Canynge Hall, 39 Whatley Road, Bristol BS8 2PS, UK; 2 Centre for the Study of Choice, University of Technology Sydney, City – Haymarket Campus, Broadway NSW 2007, Sydney, Australia; 3 Department of Community Based Medicine, University of Bristol, 25 Belgrave Road, Bristol BS8 2AA, UK; 4 Department of Health Economics, Public Health Building, University of Birmingham, Birmingham B15 2TT, UK

## Abstract

**Background:**

Additional insights into patient preferences can be gained by supplementing discrete choice experiments with best-worst choice tasks. However, there are no empirical studies illustrating the relative advantages of the various methods of analysis within a random utility framework.

**Methods:**

Multinomial and weighted least squares regression models were estimated for a discrete choice experiment. The discrete choice experiment incorporated a best-worst study and was conducted in a UK NHS dermatology context. Waiting time, expertise of doctor, convenience of attending and perceived thoroughness of care were varied across 16 hypothetical appointments. Sample level preferences were estimated for all models and differences between patient subgroups were investigated using covariate-adjusted multinomial logistic regression.

**Results:**

A high level of agreement was observed between results from the paired model (which is theoretically consistent with the 'maxdiff' choice model) and the marginal model (which is only an approximation to it). Adjusting for covariates showed that patients who felt particularly affected by their skin condition during the previous week displayed extreme preference for short/no waiting time and were less concerned about other aspects of the appointment. Higher levels of educational attainment were associated with larger differences in utility between the levels of all attributes, although the attributes *per se *had the same impact upon choices as those with lower levels of attainment. The study also demonstrated the high levels of agreement between summary analyses using weighted least squares and estimates from multinomial models.

**Conclusion:**

Robust policy-relevant information on preferences can be obtained from discrete choice experiments incorporating best-worst questions with relatively small sample sizes. The separation of the effects due to attribute impact from the position of levels on the latent utility scale is not possible using traditional discrete choice experiments. This separation is important because health policies to change the levels of attributes in health care may be very different from those aiming to change the attribute impact *per se*. The good approximation of summary analyses to the multinomial model is a useful finding, because weighted least squares choice totals give better insights into the choice model and promote greater familiarity with the preference data.

## Background

Discrete choice experiments elicit people's preferences for goods or services based on intentions expressed in hypothetical situations [1]. In traditional discrete choice experiments – also called (choice-based) conjoint analysis by many practitioners in North America – respondents choose the most preferred specification of a good ('alternative' or 'profile') from a choice set of competing attribute profiles [2]. When respondents choose their preferred profile, they effectively provide information about their preferences *relative *to some reference profile. Consequently, the utility estimates represent a set of deviations that cannot be used directly to make statements about the *overall *impact of attributes [3].

Certain issues in Health Services Research require analysts to compare the overall impact of attributes. An example would be investigating the contention that "waiting time for an appointment is more important to patients than continuity of care" – a statement purely about attributes, with no reference to the associated levels. The issue of separating attribute impact weights and scales is essentially equivalent to estimating the utility associated with a particular attribute per se (its weight or impact in a utility function) separately from the additional utility gained/taken away by that attribute exhibiting an attractive/unattractive level (the utility level scale value). This issue has been explored and several methods now are available to help address it in the context of traditional choice experiments [4].

Best-worst scaling [5], developed by Finn and Louviere [6] and introduced to health care research by McIntosh and Louviere [7] is a solution that utilizes a different design of choice experiment. A guide to using best-worst scaling is available [3], but briefly, unlike most traditional discrete choice experiments, best-worst scaling presents respondents with profiles (in this case appointments) one at a time. Respondents make choices within profiles (appointments) rather than between profiles. Thus, for a given profile, the set of alternatives on offer comprises the attribute levels that define that particular profile (appointment). By choosing the best and worst attribute levels on offer within that profile, respondents select that pair (best and worst) of attribute levels that lie furthest apart on the latent utility scale. Thus, variations on best-worst scaling have appeared, sometimes called "maximum difference scaling[8].

Various methods of analysis of best-worst choice data in a random utility framework have been described [3]. Because the data used in that illustration came from a pilot quality of life study, there is a need to investigate the empirical properties of best-worst scaling methods using a larger study. In particular, interest in investigating preference heterogeneity is growing [9,10], so there is a need to illustrate the considerable insights that can be gained from analyses of best-worst scaling choice data.

The additional information provided by best-worst scaling compared with a traditional choice model is useful to the researcher in three ways.

1. Most importantly, asking about worst as well as best elicits more information about the respondent's utility function. This increases the researcher's ability to characterise heterogeneity in preferences and classify respondents into internally homogeneous groups. In certain circumstances best-worst methods can even be used to estimate individual-level utilities [11].

2. For a given attribute, all of the level scale values are estimated (rather than all but one as in a traditional choice experiment) [3]. This allows the researcher to calculate the mean utility across an attribute's levels – the attribute impact. Furthermore, all attribute levels (across the entire study) are estimated on a common scale, allowing meaningful comparisons of attribute impacts to be made. However, it should be noted that attribute impact is not the same as attribute importance – a more generalisable concept that has been investigated (largely unsuccessfully) by psychologists for 40 years [12]. Nevertheless, because all the level scale values (and hence all the attribute impacts) are on a common scale, knowledge of an attribute's overall impact is still useful. It may help policy-makers decide whether policies to improve levels of key attributes (for instance reduce the incidence of a given side effect of treatment) or those to increase/decrease the perceived impact of attributes themselves (for instance better education to improve patient understanding of a side effect) are the most desirable or feasible.

3. When effects coding (rather than a dummy variable approach) is used to estimate the level scale values: the econometric model automatically estimates the statistical significance of both level scales and the overall attribute impact.

A second issue worthy of exposition concerns the choice of regression model. Four models have been proposed for the analysis of best-worst scaling data in a random utility framework [3] – the researcher can choose between analysing at the level of the best-worst pair or the attribute level and between a multinomial model or a weighted least squares one. No guidance has (before now) been provided as to the relevant merits of the paired and attribute level formulations. Furthermore, practitioners of discrete choice experiments may not recognise the value of weighted least squares-based analyses which, by utilising a dataset of means/totals, promote greater familiarity with the data and thereby address the possible criticism that the methods are a 'black box'.

Therefore this paper has two aims:

1. to illustrate, for the first time in a health or medical context, the ability of best-worst scaling to estimate sub-group preferences in terms of differences in both attribute impacts and level scale values,

2. to offer recommendations on choice of analytic model and in particular to demonstrate the validity of least squares methods for aggregated analyses

The paper addresses these issues and demonstrates the flexibility of best-worst scaling methods in unbalanced designs (where the number of levels per attribute is not constant). Best-worst scaling methods, including a summary of the empirical case study, will be described first, followed by the results. The paper will conclude with the implications of this work.

## Methods

The empirical work was undertaken in the context of a project aimed at quantifying preferences for different aspects of access to dermatology secondary care services [13]. The discrete choice experiment was conducted alongside a randomised controlled trial, with associated economic evaluation; it compared consultant-led out-patient care with local care provided by a General Practitioner with a Special Interest in Dermatology [14,15]. Identification of attributes and levels was accomplished via extensive qualitative work, [16] ensuring that the attributes were relevant and grounded in patients' experiences. Four attributes were identified: waiting time, degree of expertise of doctor, convenience of attending and perceived thoroughness of care. Waiting time had four levels whilst the other attributes all had two levels, as shown in Table 1.

**Table 1:** Attributes and attribute levels

Attribute	Levels
Time waited	You will have to wait ***three months*** for your appointment
	You will have to wait ***two months*** for your appointment
	You will have to wait ***one month*** for your appointment
	Your appointment will be ***this week***

Expertise	The specialist has been treating skin complaints part-time for 1–2 years
	The specialist is in a team led by an expert who has been treating skin complaints full-time for at least 5 years

Convenience	Getting to your appointment will be difficult and time-consuming
	Getting to your appointment will be quick and easy

Thorough care	The consultation will ***not*** be as thorough as you would like
	The consultation will be as thorough as you would like

**Figure 1:**
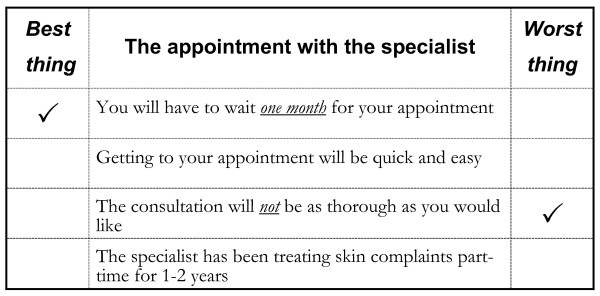
example best-worst scaling question from the study.

Construction of an appropriate design is described elsewhere, [17] and two versions of the questionnaire were created, a long version that used 16 profiles and a short version that used eight. The analysis reported here relates to the long version. In each profile (appointment offered) respondents were asked to choose one attribute that was best and one that was worst, based on the levels described in the profile. Thus, each choice represented a pair of attribute levels (see Figure 1).

Best-worst scaling data can be analysed in many ways [5]. In the random utility model formulation [3], choice data can be aggregated (or not) across attribute level pairs and/or across respondents. Paired analysis models treat each unique best-worst pair as an outcome (the 'maxdiff' model [5]) whilst marginal analysis models treat each attribute level as an outcome (aggregating pairs up to give the marginal frequencies). Weighted least squares is an appropriate way to estimate conditional logit models when choices are aggregated across respondents [18], whilst maximum likelihood is more appropriate when respondent level inference is required. The degree of aggregation across choices – whether at the paired or marginal level – does not have implications for analysis method. A full exposition of these methods has been given before [3] so the next sections will summarise them briefly with reference to this study in particular.

### Paired model conditional logit analysis

The paired method of analysis treats each unique best-worst pair as a unique choice outcome (where order matters). In a design with *K *attributes where *n*_*k *_represents the number of levels of attribute *k*, the total number of possible outcomes (pairs) in a main effects design is therefore 2 $\sum_{i=1}^{K-1}\left[n_{i}\sum_{k=i+1}^{K}n_{k}\right]$, or 72 unique pairs in the case of this study. The main effects designs utilised ensured that across all profiles in the best-worst exercise the utility difference in every one of the pairs could be estimated. Conditional (multinomial) regression models were estimated from the data with the *clogit *command in Stata with cluster-adjusted (robust) standard errors [19]. This required the data to be manipulated to ensure it was in the correct format: each chosen outcome was expanded into as many outcomes as were available to be chosen in that choice set (i.e., K(K-1) = 12 pairs). The dependent variable was coded equal to one for a chosen outcome, and coded equal to zero for all remaining (non-chosen) pairs in a particular choice set for each individual. In the case of coding qualitative attributes in discrete choice experiments, the benefits of effects coding and possible problems with traditional (1,0) dummy coding have recently been illustrated in health economics [20,21]. Effects coding is particularly well suited to best-worst scaling because attribute impacts are estimated separately from the utility level scale values (deviations from mean utility – the attribute impact), allowing both comparisons of attribute impact and significance of level scale values to be estimated directly.

The equation below sets out the relationship between the difference in utility between best and worst ($U_{diff}^{i}$ on the latent utility scale) for choice *i *(*i *= 1,2,...,12) and the nine independent variables: three estimated impact and six estimated level scale parameters:

$$\begin{array}{l} U_{diff}^{i}=\beta_{dr}D_{dr}^{i}+\beta_{conv}D_{conv}^{i}+\beta_{thoro}D_{thoro}^{i}+\\ \beta_{3m}D_{3m}^{i}+\beta_{2m}D_{2m}^{i}+\beta_{1m}D_{1m}^{i}+\beta_{pt}D_{pt}^{i}+\beta_{hard}D_{hard}^{i}+\beta_{notthoro}D_{notthoro}^{i}+\varepsilon^{i} \end{array}$$

Thus for choice *i*, the attribute chosen as best had its impact variable ($D_{attribute}^{i}$) taking value one, that chosen as worst had its impact variable taking value minus one, with the third impact variable taking value zero. (The impact for waiting time was the omitted variable and represents the zero on the utility scale). The six level scale values (respectively three, one, one and one for waiting time, doctor expertise, convenience and thoroughness) were effects coded. As for the impact variables, when an attribute level was picked as worst, its sign was reversed. The standard conditional logistic function links the observed discrete choice (zero or one) with the estimated latent utility.

### Marginal model conditional logit analysis

The marginal model aggregates the choice data to estimate the attribute level utilities using a model that is simpler, at least for main effects designs like the one illustrated here. However, this model is actually an approximation to the paired (sometimes also called 'maxdiff') model of choices. In particular, it assumes that for a given pair of attribute levels, the difference in utility simply changes sign when the best-worst choice is reversed. This can only be true if the distribution of the error term on the latent utility scale is symmetrical [22]: the gumbel distribution that underpins all logistic models is asymmetrical (although almost symmetrical). Thus, the multinomial logit marginal model should not be regarded as a gold standard (although the multinomial probit does not suffer from the asymmetry problem). Conditional (multinomial) regression was used to estimate respondent-level utilities. Each chosen attribute level was expanded to the 2K = 8 attribute levels (4 best and 4 worst) available in each choice set (profile). The outcome variable is coded equal to one for the chosen outcome (whether a best attribute level or a worst attribute level) and equal to zero for the remaining (non-chosen) attribute levels for a particular profile (appointment) and individual. The independent variables were coded with a sign change for all observations pertinent to the worst choice data, to reflect the reciprocal relationship between best and worst probabilities. It should be noted that any inference using the log-likelihood from the marginal method is potentially misleading, since the likelihood function assumes best and worst choices are made independently.

### Effects of respondent-level covariates

One aim of the study was to estimate the effect of respondent characteristics (e.g., age and clinical factors) on utilities, which requires respondent-level choice data. Limited dependent variable models require differences in the probabilities of choice for the various outcomes in a choice set to be associated with differences in the explanatory variables. Respondent characteristics like age do not vary for potential best-worst pairs or attribute levels in a choice set; hence, they cannot affect choice probabilities and cannot be separated from the overall regression constant term. Thus, covariates are interacted with choice outcomes (attribute impacts and level scales). These took the form of covariate-attribute impact interactions and covariate-level scale interactions. The former represented the additional impact a given attribute had upon the particular clinical-sociodemographic group whilst the latter represented the additional level scale utility derived by the particular patient group.

Given that the marginal model is an approximation to the paired (maxdiff) choice model, inference about the effects of respondent-level covariates on preference is reported for the paired model. However, major differences that arose from using the marginal model also are reported. In terms of sample size, given that the original trial was not powered to detect differences between subgroups, it was decided to be conservative in all analyses and reporting. Therefore, respondents who did not provide complete choice and socioeconomic data were omitted. A series of regressions which partially adjusted for each of the socioeconomic/clinical factors listed below were conducted:

• Age 60+

• Left fulltime education at age 19+

• In fulltime employment

• Reporting severe problems on at least one item of the DLQI skin quality of life scale in the previous week

• Being an 'acute' case (having first seen their GP within the previous 6 months and only seeing him/her once or twice before referral)

• Being a 'chronic' case (having first seen their GP over a year previously)

Influential covariates were then entered into a fully adjusted model. Given that best-worst scaling distinguishes between two types of preference – the attribute impact and the level scale values – statistically significant effects for either are reported.

### Comparison with least squares estimates

Discrete choice experiments in health economics usually have been analysed using limited dependent variable models like probit regression. Using weighted least squares regression on the (logged) choice frequencies would leave few, if any, degrees of freedom; for example, for the short questionnaire in this study $\sum_{k=i}^{K}\left(L_{k}-1\right)=6$ utility parameters would have to be estimated from only 8 choice frequencies. However, the additional preference information (choice frequencies) available from best-worst scaling studies makes them amenable to such methods, particularly since (well-designed) stated preference studies do not suffer from problems of multicollinearity common to revealed preference econometric studies and many epidemiological cohort studies. This makes weighted least squares useful for researchers interested in sample level (or by extension, population level) inferences because many standard statistical packages are not designed for choice models and require researchers to perform a certain amount of data manipulation; and datasets for individual level analyses can be very large, particularly in a best-worst context. Thus, it is desirable to show that orthogonal designs allow researchers to use results from weighted least squares estimation with confidence. Performing weighted least squares analysis also is useful for promoting familiarity with the data – the choice totals for all attribute levels/pairs are clear to the researcher, thus, making the procedure less open to criticisms of it being a 'black box'. So, data were analysed using weighted least squares (weights are the choice totals adjusted to eliminate sampling zeros by adding one over the 'effective' sample size[23], which is the sample size multiplied by the number of times the pair/level was available). Estimates were compared with those from maximum likelihood estimation and graphed.

## Results

Of the 119 individuals who received the 16-appointment questionnaire, 93 provided best-worst data that allowed estimation using any of the methods and 60 individuals provided complete best-worst choice data. Five of the latter individuals did not provide complete socioeconomic information; so, to be conservative, all analyses reported below pertain to the 55 who provided complete data. The minimum number of appointments answered was five whilst 85 individuals answered 14, 15 or 16 appointments. Stata chooses an attribute impact variable arbitrarily to drop in order to prevent the model being saturated. Therefore, once the least valued attribute was identified, all analyses were performed with this attribute impact omitted to ease interpretation.

### Paired model conditional logit analysis

Table 2 shows the conditional logit results using the paired method for the 55 respondents who provided complete choice and socioeconomic data.

**Table 2:** Paired model conditional logit estimates

	**Coefficient**	**Standard** **error**	**95%** **Confidence** **interval**	
**Attribute impacts**				
Waiting time	-	-	-	-
Doctor	1.3687	0.2011	0.9745	1.7628
Convenience	0.5060	0.1182	0.2743	0.7377
Thoroughness	0.3710	0.1358	0.1048	0.6372

**Level scale values**				
3 month wait	-1.4155	0.1950	-1.7977	-1.0333
2 month wait	-0.8789	0.1347	-1.1428	-0.6150
1 month wait	0.1966	0.1504	-0.0981	0.4913
No wait	2.0978	-	-	-
Part-time doctor	-1.3667	0.1331	-1.6275	-1.1059
Full-time doctor	1.3667	-	-	-
Difficult to attend	-1.0175	0.1365	-1.2851	-0.7499
Easy to attend	1.0175	-	-	-
Not thorough	-2.5347	0.2677	-3.0594	-2.0100
Thorough	2.5347	-	-	-

Log pseudolikelihood = -1246.9491

Waiting time was the attribute with least impact and its impact weight is therefore omitted – impact figures for the other three attributes therefore are relative to waiting time (which is the zero on an interval scale). Doctor expertise is clearly the most highly valued attribute, whilst convenience is valued slightly more than thoroughness of care. The result of separating overall attribute impact from level scale values is clear: whilst thoroughness of care is not the most important attribute per se, the two levels are very far apart on the utility scale. In contrast, for convenience of attending there is a difference of 2 × 2.53 = 5.06 units between the levels of thoroughness of care but only 2 × 1.02 = 2.04 units between the levels of convenience. This illustrates a key advantage of best-worst scaling over traditional discrete choice experiments: in the latter, only these differences between the levels are estimable.

### Marginal model conditional logit analysis

Table 3 shows the conditional logit results using the marginal method for the 55 respondents who provided complete data.

**Table 3:** Marginal model conditional logit estimates

	**Coefficient**	**Standard** **error**	**95%** **Confidence** **interval**	
**Attribute impacts**				
Waiting time	-	-	-	-
Doctor	1.4614	0.1878	1.0933	1.8295
Convenience	0.5225	0.1142	0.2987	0.7463
Thoroughness	0.3096	0.1407	0.0338	0.5855

**Level scale values**				
3 month wait	-1.0897	0.1317	-1.3478	-0.8316
2 month wait	-0.6817	0.0959	-0.8696	-0.4937
1 month wait	0.1261	0.1141	-0.0975	0.3498
No wait	1.6453	-	-	-
Part-time doctor	-1.0617	0.0835	-1.2253	-0.8981
Full-time doctor	1.0617	-	-	-
Difficult to attend	-0.7833	0.0845	-0.9488	-0.6177
Easy to attend	0.7833	-	-	-
Not thorough	-2.1928	0.1802	-2.5461	-1.8396
Thorough	2.1928	-	-	-

Log pseudolikelihood = -1943.8638

**Figure 2:**
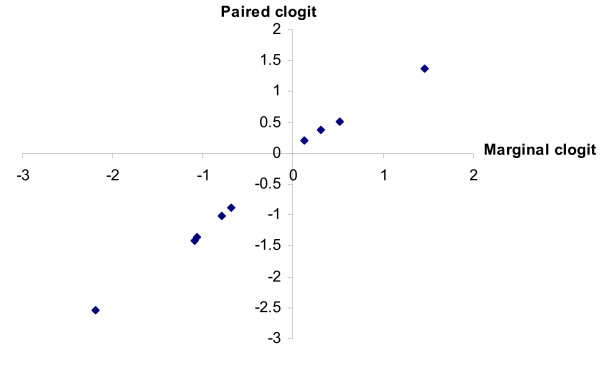
**clogit estimates; sample size = 55**. Graph of paired clogit estimates against marginal clogit estimates.

The only non-significant estimate in Table 2 is less significant in Table 3. It should be noted, however, that the log-likelihood statistic is incorrect due to the model's failure to account for non-independence of the best and worst choices in a given profile. Overall the results are qualitatively identical to those from the paired method and Figure 2 graphs the two sets of results.

### Effects of respondent-level covariates

All covariates except age and employment status had at least one statistically significant effect upon preferences. The fully adjusted model therefore includes all interactions between attribute impact weights and level scale values for the remaining covariates. In the fully adjusted model being a 'chronic' case was associated with nonsignificant effects on all preferences, so all effects related to that covariate were omitted. Table 4 summarises the paired results for the remaining three covariates for the 55 respondents who provided complete data. Seven covariate parameters were statistically significant at the 5% level. Three of these were also significant in the marginal model and the remaining four all had the same sign under both models. For the marginal model there was a fourth covariate that was significant; it had almost reached significance in the paired model.

**Table 4:** Paired model clogit estimates adjusting for covariates

	**Coefficient**	**Standard** **error**	**95%** **Confidence** **interval**	
**Attribute impacts**				
Waiting time	-	-	-	-
Doctor	1.6711	0.1841	1.3103	2.0318
Convenience	0.7154	0.1621	0.3977	1.0330
Thoroughness	0.5384	0.2040	0.1386	0.9382
Education * Doctor	-0.1326	0.2028	-0.5300	0.2648
Education * Convenience	0.0009	0.1301	-0.2541	0.2558
Education * Thoroughness	-0.0731	0.1691	-0.4046	0.2584
Severe * Doctor	0.0235	0.2233	-0.4141	0.4610
Severe * Convenience	0.0554	0.1395	-0.2180	0.3288
Severe * Thoroughness	0.0979	0.1531	-0.2021	0.3980
Acute * Doctor	0.4174	0.1801*	0.0644	0.7703
Acute * Convenience	0.2372	0.1401	-0.0373	0.5117
Acute * Thoroughness	0.2342	0.1724	-0.1038	0.5722

**Level scale values**				
3 month wait	-2.0526	0.2995	-2.6396	-1.4656
2 month wait	-1.2388	0.1984	-1.6277	-0.8499
1 month wait	-0.0402	0.2414	-0.5134	0.4330
No wait	3.3317	-	-	-
Part-time doctor	-1.7066	0.2093	-2.1168	-1.2964
Full-time doctor	1.7066	-	-	-
Difficult to attend	-1.5013	0.1832	-1.8603	-1.1423
Easy to attend	1.5013	-	-	-
Not thorough	-3.5450	0.2918	-4.1170	-2.9730
Thorough	3.5450	-	-	-
Education * 3 month wait	-0.4842	0.2404*	-0.9554	-0.0130
Education * 2 month wait	-0.0205	0.1631	-0.3402	0.2992
Education * 1 month wait	0.3004	0.1820	-0.0564	0.6571
Education * part-time doctor	-0.0706	0.1506	-0.3657	0.2245
Education * Difficult to attend	-0.2810	0.1487	-0.5723	0.0104
Education * Not thorough	-0.6507	0.2598*	-1.1599	-0.1415
Severe * 3 month wait	-0.3876	0.2142	-0.8075	0.0323
Severe * 2 month wait	-0.3754	0.1536*	-0.6764	-0.0744
Severe * 1 month wait	-0.1030	0.1651	-0.4266	0.2207
Severe * part-time doctor	0.1305	0.1434	-0.1505	0.4116
Severe * Difficult to attend	0.1407	0.1304	-0.1148	0.3962
Severe * Not thorough	0.1055	0.2617	-0.4074	0.6183
Acute * 3 month wait	-0.2515	0.2372	-0.7164	0.2133
Acute * 2 month wait	-0.2919	0.1522	-0.5902	0.0064
Acute * 1 month wait	-0.5845	0.2142*	-1.0044	-0.1646
Acute * part-time doctor	-0.3197	0.1819	-0.6762	0.0367
Acute * Difficult to attend	-0.4408	0.1774*	-0.7885	-0.0931
Acute * Not thorough	-0.8625	0.2760*	-1.4034	-0.3215

* Indicates significant at the 5% level.

Log pseudolikelihood = -1162.2742

Those who left higher education at age 19 or older exhibited 'widening' of the level scale values for all four attributes, although this did not reach significance at the 5% level for doctor expertise and convenience, but the attribute impacts were not significantly different on average. A possible explanation for this result concerns the association often observed between educational attainment and social class and/or income. Thus, it might be expected that greater access to alternative sources of health care (e.g. private health care) might mean that respondents with higher education experience greater differences in utility associated with levels of the attributes.

Those with at least one of the ten factors severely disrupted by their skin condition or those with a total score of seven or more out of 30 on the DLQI [24] (indicating 'severe' problems as judged by consultants) might be expected to exhibit smaller differences between levels of attributes and possibly attenuation of differences in attribute impact weights as 'simply getting into secondary care' becomes paramount. These two problems are highly correlated so the first one (having a score of 3 on any item) was used in analysis. Indeed waiting at all gave this group more disutility than the average, although a two month wait was the only level to reach significance at the 5% level. Although not significant, the additional utility this group experienced over the average for the lower levels of the three non-waiting time attributes was positive; that is, they did not experience as large a difference in utility between the two levels as other people.

Being an assumed 'acute' case (proxied by having first seen their GP within the previous 6 months and only seeing him/her once or twice before referral) affected preferences in various ways. Like patients with a score of three on any DLQI item, this group had a strong preference for no waiting time (demonstrated by the lower utilities associated with the three lower levels). However, they also exhibited widening of the level scale values for the other three attributes, indicating that they are more willing to trade waiting time for changes in these than those who believe aspects of their daily lives are severely affected by their skin condition. These three attributes also have a higher impact on average.

### Comparison with least squares estimates

**Figure 3:**
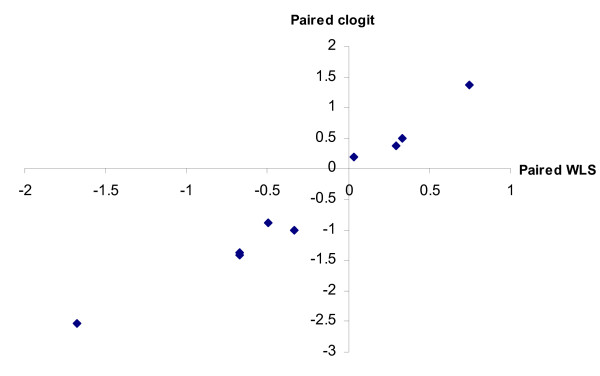
**paired estimates; sample size = 55**. Graph of paired clogit method estimates plotted against paired weighted least squares estimates.

Figure 3 shows an ordinary least squares regression of the weighted least squares estimates (estimates not shown) against the maximum likelihood estimates for the paired method for the 55 respondents who provided full information in the long questionnaire.

**Figure 4:**
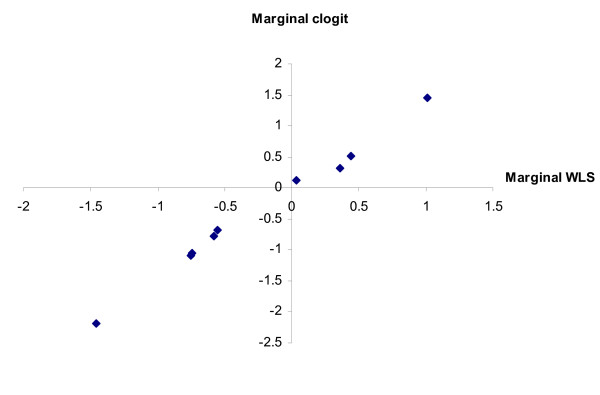
**marginal estimates; sample size = 55**. Graph of marginal clogit method estimates plotted against marginal weighted least squares estimates.

The two sets of parameter estimates are highly correlated, with R^2 ^= 0.96. The high correlation also is apparent (see Figure 4) for the marginal model analysis (R^2 ^= 0.99). This is encouraging, given that the marginal model weighted least squares regression has only 20 observations (a best and a worst frequency for each of the ten attribute levels).

## Discussion

This study is the first in health services research to separate the impact of attributes per se from their level scale values for various subgroups. It demonstrated that having self-reported severe skin problems narrows the range of scale values for non-waiting time attributes. There was a suggestion that certain socioeconomic factors, most notably leaving fulltime education at age 19+, caused the range of scale values to widen, leaving attribute impacts unchanged. Both sets of findings have intuitive explanations but given the relatively small sample they should be investigated further.

The study also provides guidance about choice of analytic model. It demonstrated that although the marginal method of analysis makes potentially unwarranted assumptions about independence of best and worst choices and the nature of the error terms in the utility model, it provides a good approximation to the paired (maxdiff) model analysis, suggesting that the resulting estimates are consistent. Furthermore, the study demonstrates the flexibility and accuracy of aggregated analysis with as few as twenty observations when factors are manipulated according to a well-conditioned design matrix. In other words, multicollinearity problems and lack of variability in key factors that necessitate large datasets in many econometric and epidemiological studies do not apply. Agreement between weighted least squares and conditional logistic regression was high, and the benefits of effects coding in a best-worst context were illustrated.

### Limitations

The sample size underlying the results was relatively small, which was due to a desire to be conservative in reporting results by excluding patients without full socio-demographic and choice data. However, similar findings were apparent (often with greater levels of statistical significance) in analyses conducted among the 93 patients who provided at least some choice data.

This study, like all discrete choice experiments, induced clustering in the data by obtaining multiple responses from individuals: two responses from the same individual tended to be more similar than two responses obtained from different individuals. Accounting for clustering via robust/sandwich/Huber-White procedures inflates the standard errors by the correlation in the error terms. Thus, it does not explicitly model heterogeneity in responses. However, the most popular approaches to modelling respondent-specific effects (random effects models) potentially are misleading. Not only do these models ignore other factors that might lead to variation in choice behaviour, virtually all published applications implicitly assumed constant error variances within and between individual responses. If the error variances do vary then the distribution of model estimates is confounded with the distribution of error variances, and the bias can be very large [25]. So, a random effects model represents at best a partial solution, and evidence suggests that this simplistic treatment of heterogeneity is not supported empirically [26]. Furthermore, unlike the case for clinical outcomes, there are no theoretically compelling reasons to support unimodal distributional assumptions for modelling preference heterogeneity.

Many discrete choice experiments in health services research suffer from designs more appropriate to traditional conjoint-analysis studies in which one tries to estimate individual-level (or small group-level) utilities rather than population parameters. In other words, using a common, small design for all respondents provides data for only a small part of the entire response surface, typically making it impossible to estimate interactions, which would provide more complete statistical information about the indirect utility function and protect the estimated parameters against unobserved effects (e.g., interactions). To an extent, this criticism can be levelled against the present study: splitting the sample into blocks and administering different versions of the questionnaire which together spanned the full factorial (32) appointments would have addressed this issue. However, practical constraints precluded such a design. In any case, future work will exploit advantages of the current design to investigate individual-level preferences.

A final issue concerns anchoring of the utility estimates. The best-worst scaling choice data provide interval-scale estimates with unknown anchor; when total utilities for each appointment are constructed and ranked, analysts cannot know at which point utility becomes positive (indicating that the respondents will choose to attend the appointment rather than not attend). This may or may not be a limitation, depending upon how one wishes to use the estimates. Planning total service provision to match demand would require unconditional demand information, not the conditional demand information that these results provide. However, marginal changes in service provision can be addressed using these results by way of calculating marginal rates of substitution as in a traditional discrete choice experiment (although the obvious lack of linearity in the utility of waiting time requires more complex calculations in this case). Constructing an outcome or service index based on these results is also possible, but more generally it may be that the need for an anchor necessitates additional information from respondents.

### Future work

One of the aims of this study was to investigate differences in response rates and results between two versions of the questionnaire – one with 8 appointments and one with 16. Differences were found to be minimal [17], so future work should compare longer questionnaires, perhaps 16 versus 32 profiles. If one uses a small design similar to the one here, such a design may permit investigation of interactions and/or any other violations of the independence of irrelevant alternatives (IIA) assumption.

It should be standard practice to adjust for clustering in responses if using covariate-adjusted conditional logit models to analyse best-worst data. However, if modelling heterogeneity explicitly, it can be argued that it is more logical to exploit the power of best-worst methods to make individual-level inferences (estimate parameters for each individual) than attempt to specify them as random effects. Indeed, best-worst scaling can model individual-level utility functions without statistically questionable distributional assumptions about preferences [11]. Similar work will be performed for these data.

The usefulness of attribute impact as a concept is an empirical issue. Because it is simply the arithmetic mean of the levels of an attribute, the impact for an attribute like thoroughness which is defined by its two 'extreme' levels (yes/no) is arguably of little value. However, more generally, attribute impact undoubtedly would be useful to researchers; for example, a non-significant scale value coupled with a very small attribute impact (relative to other attributes) may indicate that an attribute is highly disliked per se and respondents do not perceive any difference between levels. Thus, unlike traditional discrete choice experiments, researchers would know that respondents particularly dislike this attribute per se and that policies that attempt to change patient perceptions of the good/service may be more fruitful.

This study also asked respondents whether they would attend each appointment offered them. The results from the responses to this question provide an alternative set of utility estimates (although relative to one appointment or the mean utility). Differences between the two methods will be investigated as will the extent to which the anchor provided by these data can be used to rescale the best-worst data. Future studies will consider utilising qualitative work and simulation studies to ascertain whether the cognitive processes undertaken by respondents provide support for such a data synthesis.

## Conclusion

This study shows that aggregated methods provide simple compact datasets yet give results that differ little from individual-level analyses. This is a potentially useful result, and one that some applied statisticians (in both health services research and economics) may find surprising, given the multicollinearity problems common to many epidemiological and economic surveys. It also illustrates a key advantage of best-worst scaling over traditional discrete choice experiments – the ability to separate attribute impacts from level scale values. In so doing it provides additional insights over those from traditional discrete choice experiments that should prove attractive in health care research. In particular, this ability to ascertain whether patient subgroups exhibit differences in attribute impact and/or differences in level scale values may have implications for policy.

## Competing interests

The authors declare that they have no competing interests.

## Authors' contributions

TNF carried out all primary data analyses and drafted the paper. JJL designed the methodology, advised on study design and analysis and participated in redrafting of the paper. JC participated in the design and running of the study, and in redrafting of the paper. TJP performed statistical input to the study and participated in redrafting of the paper.

## Pre-publication history

The pre-publication history for this paper can be accessed here:


